# Urinary bio-monitoring of amphetamine derivatives by needle trap device packed with the zirconium-based metal–organic framework

**DOI:** 10.1038/s41598-022-17861-1

**Published:** 2022-08-11

**Authors:** Razzagh Rahimpoor, Ali Firoozichahak, Saber Alizadeh, Davood Nematollahi

**Affiliations:** 1 Department of Occupational Health Engineering, School of Health, Research Center for Health Sciences, Larestan University of Medical Sciences, Larestan, Iran; 2 Department of Occupational Health, Faculty of Health, Social Determinants of Health Research Center, Gonabad University of Medical Science, Gonabad, Iran; 3 Department of Chemistry, Bu-Ali-Sina University, Hamedan, Iran

**Keywords:** Biochemistry, Biological techniques, Developmental biology, Environmental sciences, Biomarkers, Chemistry

## Abstract

In this research, zirconium-based metal–organic framework was utilized as a novel and efficient porous adsorbent for headspace extraction of Amphetamine, Methamphetamine, and Fenfluramine from the urine samples by a needle trap device (NTD). The Zr-UiO-66-PDC was electrosynthesized at the green conditions and characterized by various analyses such as FT-IR, XRD, FE-SEM, EDS, and elemental mapping techniques. Then, the effective parameters on the NTD efficiency such as salt content, pH, extraction/desorption temperature and time were evaluated and optimized by response surface methodology. The optimal extraction of amphetamine compounds was accomplished in 50 min at 70 ºC at the situation with NaCl content of 27% and pH: 11.90. The limit of detection, and limit of quantification factors were determined to be 0.06–0.09 and 0.5–0.8 ng mL^−1^, respectively. Furthermore, the precision and accuracy (intra- and inter-day) of the employed procedure in the term of relative standard deviation (RSD) were calculated in the range of 8.0–9.0% and 6.8–9.8%, respectively. Also, the recovery percent of the extracted analytes were concluded in the range of 95.0–97.0% after 10 days from the sampling and storage at 4 °C. Finally, the proposed procedure was involved in the analysis of amphetamine compounds in the real urine samples. These results were proved the proposed Zr-UiO-66-PDC@HS-NTD technique coupled with GC-FID can be used as an eco-friendly, fast-response, sensitive, and efficient drug test procedure for trace analysis of the amphetamine compounds in urine samples.

## Introduction

The growing trend of environmental pollutants and finding eco-friendly controlling solutions include sampling, extraction, determination, and degradation or removal of them is one of the important concerns of the current generation^1,2^. Drug active materials are one of the most important sources of pollutants due to the excessive consumption of drugs and the possibility of accumulation in human and biologically viable tissues^3^. Amphetamine compounds as sympathomimetic amine drugs are known as the central nervous system stimulator and can be employed for the treatment of mild depression, obesity, narcolepsy, and some behavioral problems in children. On the other hand, these synthetic drugs as powerful neurostimulators can be caused psychotic disorders such as euphoria, hallucinations and delirium, and even death^4–6^. The most common amphetamine compounds are amphetamine, methamphetamine, and fenfluramine compounds. Due to the psychoactive properties of amphetamine compounds and their misapplication attends to find a promise and reliable analysis method for biomonitoring of these compounds is still important in toxicology, occupational medicine, and forensics medicine. One of the non-invasive analysis candidates for bio-monitoring of drugs is the employment of non-metabolized forms of them in the urine matrix^7,8^.

Different conventional methods such as liquid–liquid extraction (LLE) and solid-phase extraction (SPE) have been utilized for the extraction of these compounds in the urine matrices^9–11^. These conventional extraction methods suffer from several drawbacks such as low accuracy, multi-step, and prolonged-time process. Also, a requirement of a large volume of solvents and the loss of analytes should be considered from an economically and environmentally standpoint. Microextraction techniques as one of the modified extraction methods have been provided with the one-step, cost-effective, solvent-free, user and environmentally-friendly procedure that involved quantitatively determination of amphetamine compounds in the biological matrices^12–14^. But, most of these methods have some disadvantages such as fibre fragility, non-active sampling, and limited adsorption capacity despite the various advantages.

The needle trap device (NTD) is one of the novel samplings and microextraction methods. This device which comprises a packed needle with a suitable solid adsorbent operates based on diffusion of guest analyte in a non-equilibrium mode in the air environment or the upper space of aqueous solutions (headspace)^15^. From the eco-friendly standpoint, the NTD provides a free-organic solvent procedure with a one-step run for sampling to determination of targeted analytes. Furthermore, this method could be addressed the needle fragility problem along with the increased absorption capacity^16–18^. So, the NTD technique has been known as an efficient and environmentally friendly procedure. It should be noted that NTD as a flexible method can be adopted with the different adsorbents as a packing agent^3,19^.

One of the best porous materials as packing candidates is Metal–Organic Frameworks (MOFs) with hierarchically crystalline networks of electron-donor linkers and electron-acceptor metallic cations^20^. MOFs have been employed in various chemical and electrochemical applications due to their unique and interesting properties such as flexibility of synthesis procedures, structural diversity, tunable porosity, low density, high surface area, reusability, and suitable stability^21,22^. For example, GO-ZIF-8 has been used for the separation of amphetamine from the water^23^.

The literature review shows interesting and eye-catching studies that have been focused on the use of NTD packed MOFs for the determination of the deferent analytes in the urine samples^24,25^. To the best of our knowledge, there is not any document on the use of Zr-UiO-66-PDC (PDC: Pyridine-2,6-dicarboxylate) MOF for microextraction and determination of amphetamine derivatives from the urine samples by the HS: NTD method. Herein, the Zr-UiO-66-PDC as with high surface area, high thermal resistance, and high porosity^26,27^ were employed for sampling, microextraction and determination of the amphetamine derivatives (Amphetamine, Methamphetamine, and Fenfluramine) in the laboratory and real human urine samples by HS:NTD-GC procedure. In this way, optimization of effective extraction and desorption parameters on the NTD was accomplished by Face-Centered Central Design- response surface methodology (FCCD-RSM) software. Finally, the performance of the proposed Zr-UiO-66-PDC@NTD technique was investigated in the real workplace samples along with the evaluation of control parameters such as Limit of Detection (LOD), Limit of Quantification (LOQ), Linear Dynamic Range (LDR), carryover, storage stability, and breakthrough volume. The obtained results illustrated that the proposed procedure can be employed as a drug test for the determination of the amphetamine derivatives in employments, sports, poisoning diagnostics, and forensics.

## Materials and methods

### Chemicals and reagents

Zirconium tetrachloride (ZrCl_4_) (Sigma Aldrich, 98%), Pyridine-2,5-dicarboxylic acid (H_2_PDC) (Merck, 95%), Potassium Nitrate (KNO_3_) (Sigma-Aldrich, 99%), and Ethanol (C_2_H_5_OH) (Merck, 99%) were reagent-grade materials and consumed as received without any purification. Standard solutions of amphetamine, methamphetamine, and fenfluramine in methanol (1.0 mg mL^−1^), were purchase from Sigma-Aldrich (Busch-Switzerland). Pure water was obtained from a Milli-Q IQ 7000 Merck Millipore system (Schwalbach, Germany). All solutions were prepared at room temperature.

### Instruments

The determination of amphetamine compounds was carried on a Varian CP-3800 gas chromatography system equipped with a flame ionization detector (GC-FID), and capillary column Chrompack CP7860 column (CP-Sil 5 CB, 30 m × 0.25 mm × 0.33 µm). To separate of targeted analytes, the column temperature program was set as follow: initial temperature 50 °C (kept 2 min), ramped at 6 ºC min^−1^ to 120 °C, and held at 120 °C for 2.5 min, then increased at 8 °C min^−1^ to 280 °C and kept for 5 min. The pure nitrogen (99.999%) with a flow rate of 4.0 mL min^−1^ was used as carrier gas. The injection port and FID temperature were maintained at 200–300 °C and 280 °C, respectively. Powder X. ray diffraction measurement was carried on a Bragg − Brentano XRD (2θ, 5–80° geometry; Cu Kα: model APD 2000; Italy) equipped with Using a linear sensor (Saint-Gobain). Field-Emission Scanning Electron Microscopy (FE-SEM) images of electrosynthesized Zr-UiO-66-PDC MOF were obtained using TESCAN MIRA3. Infra-red absorption spectra were performed on an ABB FTLA 2000 Fourier transform infrared spectrometer (400–4000 cm^−1^).

### Electrosynthesis of Zr-UiO-66-PDC MOF

The Zr-UiO-66-PDC MOF was fabricated based on the previously reported documents^28^. In this electrochemical procedure, 0.366 g (2.2 mmol) of pyridine-2,5-dicarboxylic acid (H_2_PDC) as ligand 0.512 g (2.2 mmol) of zirconium tetrachloride as a cation source, and 0.127 g (0.1 mmol) potassium nitrate (KNO_3_) as a supporting electrolyte were added into the aqueous solution of formic acid (50.0 mL; H_2_O/formic acid; 1/9). Before the electrolysis (30 mA cm^−2^ for 1800s), the prepared solution was stirred for 30 min at room temperature. Electrosynthesis of Zr-UiO-66-PDC MOF was performed by applying 30.0 mA cm^−2^ for 1800s in a homemade undivided two-electrode cell. The cell consists of a cap glass bottle containing a precursor solution, carbon plate as working electrode (100 mm × 20 mm × 5 mm) and the stainless steel sheet as the auxiliary electrode (See Fig. S1). The electrosynthesis was run at room temperature and atmospheric pressure. After electrolysis, the suspension cream-colored solution was centrifuged at 5000 rpm for 5 min and the precipitate MOF powder was washed twice with ethanol and distilled water. The obtained MOF product was aged overnight at 100 °C.

### Preparation of NTD

**Figure 1:**
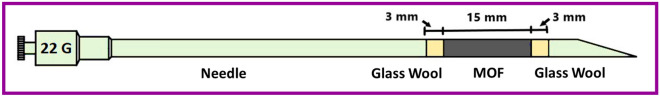
Schematic diagram of a packed needle with Zr-UiO-66-PDC adsorbent for headspace sampling of amphetamine compounds.

Spinal needles with size 22 G (90.0 mm × 0.71 mm: Tokyo, Japan) were used to prepare the packed needle with Zr-UiO-66@PDC adsorbent. The needle was filled with 2 ± 0.5 g of the mixture of the synthesized MOF adsorbent and crushed glass (in order to overcome pressure, drop and needle blockage) and was blocked by the glass wool for protection of adsorbent (see Fig. 1 and Fig. S1).

To provide the same sampling conditions for all of the NTDs, the flow rate of each needle is evaluated after the filling. In this way, the NTD is connected to the sampler pump on one side and to the port on the other side, creating conditions such as a soap bubble flowmeter. In this study, the flow rate of NTD was determined to be 3.0 ± 1.0 mL min^−1^.

### Pilot study

In a typical procedure, 10.0 mL of the solution containing the targeted analyte (30.0 μg mL^−1^) and desired amount of salt was added into a 20.0 mL glass vial and sealed. The solution pH was adjusted using NaOH 0.1 M solution on the desired value. The vial was completely closed with a Teflon/silicone and aluminum lid and placed in a water bath (for indirect heating) equipped with a glass thermometer to the monitoring of solution temperature during headspace sampling. A prepared NTD was connected on the one side at the headspace of the vial and on the other side to the sampling pump (SKC 222-3, USA) with the desired flow rate.

**Figure 2:**
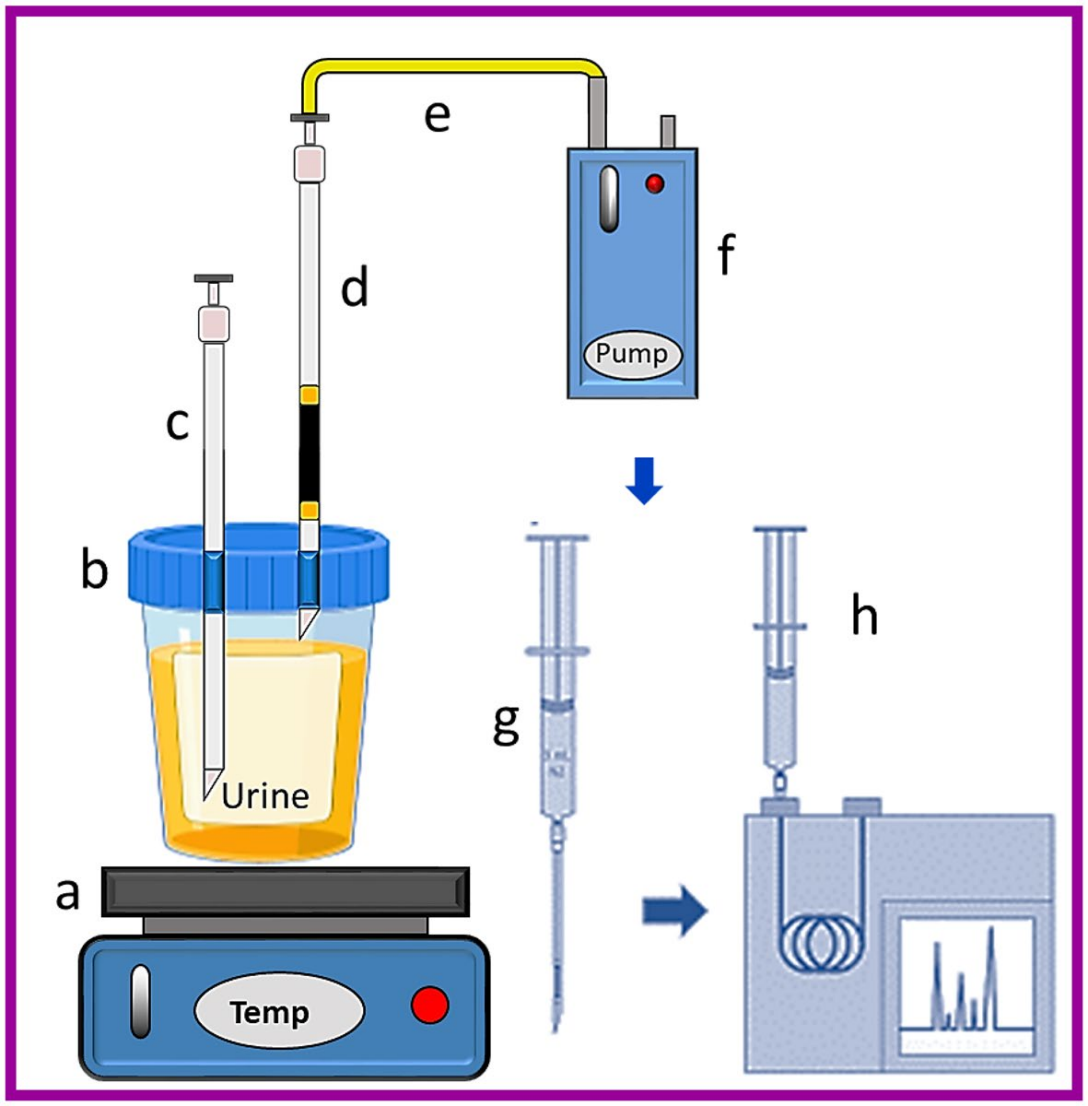
Schematic diagram of headspace sampling and analysis of the amphetamine compounds by Zr-UiO-66-PDC@NTD technique: (**a**) heater-Stirrer, (**b**) urine vial, (**c**) needle, (**d**) packed NTD, (**e**) tube, (**f**) pump, (**g**) NTD-GC needle, (**h**) GC-FID.

Also, a 22-gauge needle (adsorbent-free: blank needle) was inserted into the vial to prevent vacuum conditions during headspace sampling (Fig. 2 and Fig. S1). After headspace sampling under desired conditions, the extracted analytes were disconnected from the pump and joined to a Luer-lock syringe containing 3.0 mL nitrogen gas and immediately placed in the injection port of GC for thermal desorption (1–7 min at 200–300 °C). Thermal desorption was completed by passing 3.0 mL of nitrogen gas from the adsorbent bed containing the targeted analytes to the GC column.

### Zr-UiO-66-PDC@NTD procedure and optimization

A Face-Centered Central Design (FCCD) was applied to optimize the variables affecting the extraction and desorption steps of the studied analytes by the Zr-UiO-66-PDC@NTD method. Analysis of variance (ANOVA) at a confidence level of 95% was used to evaluate the effect of the parameters on the extraction efficiency. For this goal, extraction factors include of temperature (30–70 °C), time (15–60 min), concentration of salt (0.0–30.0 w/v %), pH value (8.0–12.0), and thermal desorption factors include of temperature (200–300 °C) and time (1–5 min) were optimized by FCCD model.

### Method validation

The validation of the proposed method was surveyed based according to IUPAC guideline^29^. To shed light on this issue, the limit of detection (LOD), the limit of quantification (LOQ), Linear dynamic range (LDR), precision (relative standard deviation %), accuracy, and extraction efficiency (%) for headspace sampling of amphetamine compounds from urine samples were investigated. To determine the LODs and LOQs of the Zr-UiO-66-PDC@NTD method for the extraction of urinary amphetamine compounds, the concentration of targeted analytes were reduced to be corresponded to the signal-to-noise ratios of 1:3 and 1:10, respectively. The precision was evaluated by intra- day and inter-day RSD. The repeatability and precision were evaluated by 6 repeat sampling and analysis of spiked three concentrations of each analyte (5.0, 15.0 and 35.0 µg mL^−1^) in one day (intra-day) and different days (inter-day) under optimized conditions and was reported as RSD%. Evaluating the accuracy was done based on the difference of the spiked and real concentrations of the analytes in the urine sample. First, three different concentrations of the analytes (5.0, 15.0 and 35.0 µg mL^−1^) were added to the blank samples, and then the extraction percentage was calculated as follows:

$${\text{R}} = \left( {\frac{{{\text{T}}_{{\text{a}}} - {\text{T}}_{{\text{b}}} }}{C} \times 100} \right)$$

where R is recovery percentage (%), also T_a_ and T_b_ are the amount of compound extracted by NTD and amount of compound desired in the blank sample, respectively. C is the concentration of spiked analyte into the sample.

Furthermore, the extraction efficiency of urinary amphetamine compounds by the proposed method was determined at three concentrations (low, medium, and high concentration: 5.0, 15.0, and 35.0 µg mL^−1^, respectively) using the following equation:

$${\text{Extraction Efficiency }}\left( {\text{\% }} \right) = \frac{{\text{C}}}{{{\text{C}}_{0} }} \times 100$$

where C and C_0_ are amounts of detected and spiked concentration of each analyte, respectively.

In this study, to investigate the carry-over effect of amphetamine compounds on the Zr-UiO-66-PDC adsorbent, the highest concentration of targeted analytes (35.0 µg mL^−1^) was sampled and analyzed based on the optimized conditions of the proposed method. The same Zr-UiO-66-PDC@NTD was injected again into the GC system after 15 min at desorption temperatures (200–300 °C) and desorption times (1–7 min).

### Real sampling

The real worked place to study was performed to the applicability of the proposed method for extracting the amphetamine compounds from real urine samples. For this purpose, the urine sample of eight methamphetamine abused suspects was collected. All experiments were performed following the general medical council Guidelines, and approved by the ethics committee at Gonabad University of Medical Sciences. Informed consent was obtained from human participants of this study. Before collecting urine samples, the workers the requested to sign the consent form. The urine sample was collected in a polyethene bottle and then was sent to the laboratory for analysis according to the optimal conditions of the proposed method. It should be noted that to calculate RSD, in each sampling step, 6 replications were performed.

## Results and discussion

### Characterization of electrosynthesized Zr-UiO-66-PDC MOF

**Figure 3:**
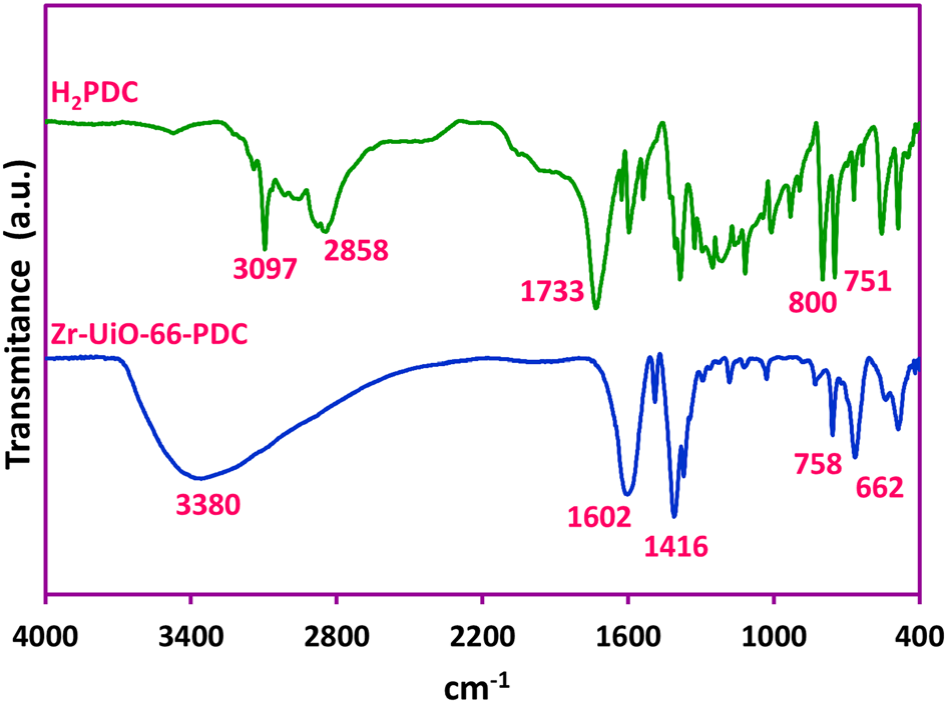
FT-IR spectra of the pyridine-2,5-dicarboxylic acid (H_2_PDC) and Zr-UiO-66-PDC samples.

Employing the green and eco-friendly techniques for preparation of chemical materials is an essential need in sustainable development^30–33^. In this research, a cathodic electrosynthesis procedure was employed for the fabrication of Zr-UiO-66-PDC MOF^34–36^ (See Fig. S1). This procedure provides a green and eco-friendly condition for the preparation of Zr-UiO-66-PDC MOF at room temperature, atmospheric pressure, and short time without the requirement for any ex-situ base/probes additive. In the following for surveying of the physicochemical properties of the prepared Zr-UiO-66-PDC the FT-IR, PXRD, FE-SEM, EDX and mapping analysis were performed. In the first step, the FT-IR analysis fabricated Zr-UiO-66-PDC MOF was done to investigate the present functional and bonding groups at the product structure. Figure 3 shows the FT-IR spectra of the used ligand (H_2_PDC) and electrosynthesized Zr-UiO-66-PDC MOF. As can be seen, the recorded spectrum is matchable with the previously reported documents^37^. The disappearance of peaks at 3098–2858 cm^−1^ and a free carbonyl group peak at the 1733 cm^−1^ in the Zr-UiO-66-PDC MOF spectrum can be assigned to the contribution of the Pyridine-2,5-dicarboxylic acid (H_2_PDC) in the configuration of the final MOF structure. Furthermore, the coordination of the H_2_PDC as employed ligand with the Zr^4+^ as available cation can be diagnosed by the presence of asymmetric and symmetric vibration coupled peaks at 1602–1416 cm^−1^ in the Zr-UiO-66-PDC spectrum.

**Figure 4:**
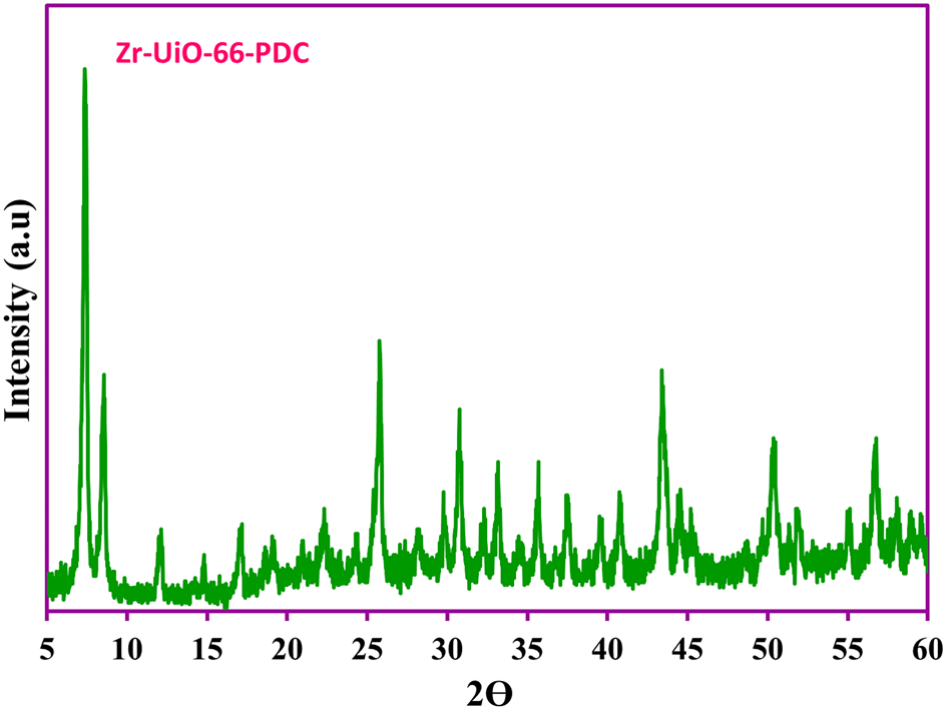
PXRD spectrum of the Zr-UiO-66-PDC MOF sample.

Also, the crystallinity structure and purity of the prepared Zr-UiO-66-PDC absorbent was evaluated by the PXRD analysis. The characterized diffraction peaks at the obtained pattern exhibited the purity and suitable crystallinity structure of the prepared MOF which is compatible with the previously reported^36^ (Fig. 4).

**Figure 5:**
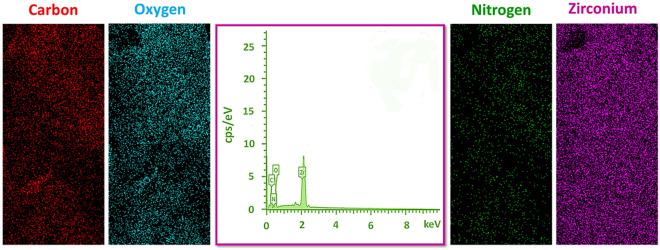
EDX diagram and elemental mapping of the Zr-UiO-66-PDC MOF.

In the following, the EDX and mapping analysis was accomplished for verification of the composing ingredients in the Zr-UiO-66-PDC structure. (Fig. 5). The obtained elemental pattern indicated the simultaneous cooperation of Zr, N, C, and O as mixed up elements in the Zr-UiO-66-PDC arrangement. Furthermore, the recorded elemental mapping illustrated the suitable, homogeneous, and uniform presence and distribution of Zirconium, Nitrogen, Carbon, and Oxygen elements in the Zr-UiO-66-PDC MOF.

**Figure 6:**
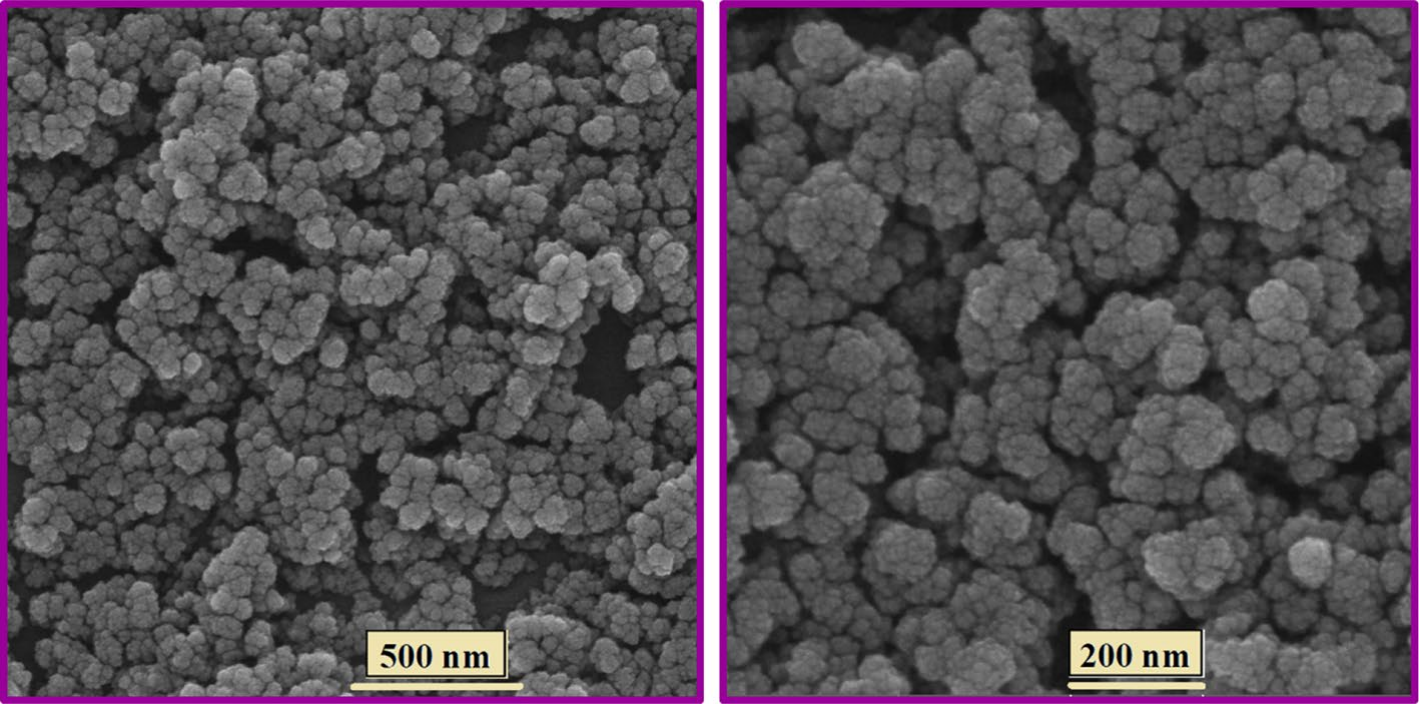
FE-SEM images of the Zr-UiO-66-PDC MOF.

Finally, the surface morphological of electrosynthesized Zr-UiO-66-PDC crystals were captured by the FE-SEM technique. Figure 6 illustrates the uniform and homogeneous cauliflower-shaped nanoparticles with an average diameter size of around 25.0 nm, approximately.

### Extraction parameters

To determine the optimal extraction condition of the amphetamine derivatives from the urine samples, the effect of key parameters (such as temperature, time, pH, and salt content) and their interaction with each other on the extraction efficiency were investigated. In this regard, the response surface methodology (RSM) and Design-Expert software were used to optimize these parameters. Also, the quadratic model was used as the best model with the highest regression (R^2^) to modelling the data.

According to the findings, the highest extraction efficiency of amphetamine derivatives was obtained at extraction temperature of 70 °C, extraction time of 60 min, pH of 11.9, and salt content of 27.0 w/v % (See Table 1). Figure S2 present the interaction of parameters affecting the extraction efficiency of urinary amphetamine compounds by the Zr-UiO-66-PDC@NTD method.

**Table 1:** Optimal conditions of extraction and thermal desorption parameters of amphetamine compounds by the proposed method.

Parameters	Amphetamine	Methamphetamine	Fenfluramine
Extraction Temperature (°C)	69.6	67.9	67.0
Extraction time (min)	46.1	49.1	49.7
Salt additive (w/v %)	27.5	27.9	27.5
pH value	11.9	11.9	11.9
Desorption Temperature (°C)	275.0	277.0	280.0
Desorption time (min)	6.36	6.32	6.11

In this research, extraction temperatures of 30–80 °C were investigated. It was expected that extraction efficiency could be increased with the extraction temperature. But the obtained results indicated that higher temperatures lead to the reduced extraction efficiency of analytes. So, the best results were observed at 70 °C. It is noteworthy that the transfer rate of analyte from the liquid matrix to the upper gaseous space of the matrix can be increased by the increasing extraction temperature. This process concludes in enhanced extraction efficiency through the increasing probability of the analyte-adsorbent interaction in the NTD. On the other hand, the higher temperature can be caused by the desorption and release of the trapped analyte (due to the higher mobility of analyte molecules) during the headspace sampling process^38^. It should be noted that water evaporated molecules can be accumulated and re-placed in the adsorbent cavities, which can be reduced the adsorbent capacity and efficiency^38^. Therefore, the optimum extraction temperature was selected to be 70 °C.

The extraction time is one of the effective parameters in the study of analyte extraction from the liquid phase. At the shorter extraction times, the extraction efficiency can be decreased because of the slower transferred rate of targeted analytes from the liquid matrix to the upper space of the vial. In this regard, the effect of extraction time on the extraction efficiency was evaluated in 15–60 min that the optimal extraction time by the synthesized adsorbent for urinary amphetamine compounds was recorded at 50 min. Therefore, according to the obtained optimum extraction temperature (70 °C), this time (50 min) was considered as optimal extraction time.

In this study, the effect of NaCl additive on the extraction efficiency was investigated at three levels of 0.0–30.0% w/v. The addition of salt increases the ionic strength of the aqueous solution and therefore affects the solubility of the analyte. This parameter also plays an important role in the analyte distribution coefficient between the adsorbent and the solvent. The obtained results indicated that the extraction efficiency of amphetamine compounds in the urine samples enhanced with rising of the salt concentration. This phenomenon is derived from the fact that the possibility of transferring the rate of analytes with a lower distribution coefficient from the liquid to the gas phase can be enhanced by increasing the salt concentration in the liquid phase^39–41^. In this study, the best extraction efficiency of amphetamine compounds from the urine samples by the Zr-UiO-66-PDC@NTD method was obtained by adding 27.0% w/v salt to the aqueous sample.

In the Zr-UiO-66-PDC@NTD method, the volatility of the analytes should be considered especially for the ionized analytes. Therefore, the pH value of the sample should be adjusted so that the analytes evaporate during the extraction process. As the results show, the highest extraction efficiency of amphetamine compounds from the urine samples was obtained at pH 11.9. At this pH condition, the studied compounds are in the neutral state, and due to the increased transfer rate of analyte from the liquid sample to the gaseous phase of upper space, the extraction efficiency has increased. The results of optimization of extraction parameters are consistent with similar studies in this scientific field^39–44^. For example, in the study Nikolaos Raikos et al.^43^, extraction time and temperature were set at 60 min and 90 °C, respectively. Also in another study, pH, extraction time and temperature were set at 11.5, 50 min and 62 °C, respectively^38^. The results of ANOVA analysis in RSM methodology to optimize the extraction process of amphetamine compounds with the headspace: UiO-66@PDC: NTD method is shown in Tables S5–S6.

### Desorption parameters

In the thermal desorption process of absorbed analytes from the adsorbent by GC, time and temperature parameters can be directly affected by amounts of desorbed analytes from the adsorbent and the amount of reminded analytes in the adsorbent (carry-over). Therefore, in this study, desorption temperature (200–300 °C) and desorption time (1–7 min) were investigated and were optimized using the FCCD methodology of the design expert software. Higher desorption temperatures can reduce desorption time, but very high temperatures can easily degrade the sensitive analytes as well as reduce the useful life of the adsorbent^38^. The results showed that the optimal points of desorption parameters were in the range of 275–280 °C and 6–6.5 min, respectively (Table 1), which is consistent with other studies in this field^42,43^. For example, in the study by Shokrollahi et al.^38^, the desorption temperature and time were estimated at 280 °C and 15 s, respectively.

Figure S3 illustrated the interactive effect of desorption temperature and time on the NTD performance. According to the results, increasing the desorption temperature and time to a certain extent could be enhanced the performance of the NTD sampler. While the further increase in desorption temperature and time could be reduced the thermal desorption performance of the studied analytes. Tables S1–S3 present the analysis results of the ANOVA obtained from FCCD methodology to optimize the affecting thermal desorption parameters of amphetamine compounds sampled with the Zr-UiO-66-PDC@NTD method.

### Method validation

The carry-over effect is a well-known problem in the development of micro-extraction methods, which indicates the amount of reminded analyte on the adsorbent bed after the desorption process. In the NTD method, desorption temperature and time parameters can be affected by the carry-over effect. In order to remove the carry-over effect, Shokrollahi et al.^38^ have suggested desorption temperature and time at 280 °C and 15 s, respectively. In a previous study, SPME-coated with polydimethylsiloxane adsorbent was used to headspace extraction of the amphetamine compound from the urine samples, which any carry-over effect was not observed at 220 °C for 1 min^43^. In the presented research, the carry-over effect was investigated at different desorption temperatures and times (30.0 µg mL^−1^). The results illustrated that the carry-over effect was less than 1.0% for all of the studied analytes at a desorption temperature of 280 °C and desorption time of 6.5 min. Therefore, this condition was considered as optimal thermal desorption conditions to prevent the carry-over effect in the proposed method.

Also, the results showed that the LOD, LOQ and linear concentration range values of the Zr-UiO-66-PDC@NTD method in the extraction of urinary amphetamine compounds are in the range of 0.06–0.09, 0.5–0.8 and 0.5–40.0 ng mL^−1^ respectively. Table 2 presents a comparison of the Zr-UiO-66-PDC@NTD performance with the other previous studies for headspace extracting and analysis of the amphetamine compounds from the urine matrix. As can be seen, the proposed method has provided better or comparable results for extracting and analyzing urinary amphetamine compounds compared to other previous methods. So, the Zr-UiO-66-PDC@NTD method can be used as a sensitive, user-friendly, eco-friendly, reusable, and cost-effective method for determining trace levels of amphetamine compounds in urine samples.

**Table 2:** Comparison of Zr-UiO-66-PDC@NTD with other techniques for determination of amphetamine compounds.

Technique	Determination	Matrix	LOD^a^	LOQ^a^	RSD (%)	References
HS-SPME	GC- FID	Urine	0.10	0.9	2.8–4.4	^38^
HS-SPME	GC- MS	Urine	0.60	–	5.0	^45^
HS-SPME	GC–MS	Urine	3.00	10.0	0.5–7.6	^46^
HS-SPME	GC–MS	Urine	0.30	1.0	1.5–2.8	^47^
SPME	GC–MS	Urine	10.00	–	6.6	^48^
SPME	GC–MS	Plasma	1.00–1.50	5.0	7–1	^49^
SPME	GC–MS	Urine	0.03	0.3	0.9–10	^39^
SPME	GC–MS	Urine	0.20–1.30	0.7–2.7	2.3–9.8	^50^
HS-NTD	GC- FID	Urine	0.06–0.09	0.5–0.8	3.1–5.1	Current study

^a^ng mL^−1^.

HS, headspace; SPME, solid-phase micro-extraction; NTD, needle trap device; GC, gas chromatography; FID, flame ionization detector; MS, mass spectrometry; LOD, limit of detection; LOQ, limit of quantification; RSD, relative standard deviation.

The repeatability of the proposed method was surveyed for determining precision by calculation of the RSD% at different concentrations (5.0, 15.0 and 35.0 μg mL^−1^) on an intra- and inter-day basis with 6 replicate (Table 3). The RSD % of the proposed method for determining amphetamine compounds in the urine samples was obtained between 1.9 and 5.6%, which indicates the acceptable precision for a developed sampling and analysis technique. Table 2 shows the comparison of this study with the other performed analysis to determine the concentration of the amphetamine compounds from the urine samples. The close of the results to each other and the low RSD % in different concentrations during different days is one of the advantages of this method, which indicates the high precision of the Zr-UiO-66-PDC@NTD method in determining urinary amphetamine compounds.

**Table 3:** Precision (RSD %) and accuracy of NTD packed with Zr-UiO-66-PDC@NTD adsorbent for determining amphetamine compounds in the urine samples.

Analytes	RSD% for an NTD at different concentrations						Accuracy		
	Intra-day			Inter-day					
	5.0 µg mL^−1^	15.0 µg mL^−1^	35.0 µg mL^−1^	5.0 µg mL^−1^	15.0 µg mL^−1^	35.0 µg mL^−1^	5.0 µg mL^−1^	15.0 µg mL^−1^	35.0 µg mL^−1^
Fenfluramine	2.2	3.3	3.5	5.3	4.8	5.1	− 6.8	− 7.6	− 6.9
Methamphetamine	3.1	3.2	3.1	4.6	3.9	4.2	− 9.3	− 9.2	− 9.8
Amphetamine	3.4	3.1	2.9	4.3	4.1	4.6	− 8.4	− 7.9	− 8.3

The accuracy of an analytical method indicates the difference between the measured and initial concentration analytes. The accuracy of the Zr-UiO-66-PDC@NTD method in the determination of urinary amphetamine compounds at concentrations of 5.0, 15.0 and 35.0 μg mL^−1^ is shown in Table 3. The low difference between the obtained and the real concentrations indicates the high accuracy of the Zr-UiO-66-PDC@NTD method in determining urinary amphetamine compounds.

Furthermore, to determine the extraction efficiency of amphetamine compounds using the proposed Zr-UiO-66-PDC@NTD method, the average extraction efficiency for each analyte at three different concentrations (low, medium and high: 5.0, 15.0, and 35.0 μg mL^−1^) in the urine matrix was calculated. The average extraction efficiency of amphetamine, methamphetamine, and fenfluramine with the proposed Zr-UiO-66-PDC@NTD method was 92.8%, 93.4%, and 92.9%, respectively, which are comparable to other studies^44,46–48^.

### Real sample analysis

After optimizing the extraction and thermal desorption parameters in the laboratory, the proposed method was employed to extract and analysis of amphetamine compounds (with 6 repetitions) from the urine samples of 8 methamphetamine abused suspects. Also, urine samples of people without exposure to methamphetamine were collected and used as blank urine samples. The concentration of methamphetamine in real urine samples was estimated in the range of 18.6 ± 3.9 μg L^−1^.

Also, Fig. S4 shows the obtained chromatogram of the extracted amphetamine compounds in the headspace of a real urine sample by Zr-UiO-66-PDC@NTD followed by the GC-FID technique.

## Conclusion

The Zr-UiO-66-PDC MOF was electrosynthesized at the green and eco-friendly condition and employed for the bio-monitoring of amphetamine compounds from the urine samples by headspace NTD technique, for the first time. The sampling, microextraction, and determination of amphetamine compounds were satisfying performed by the proposed procedure as a promising technique in the laboratory and real-time samples. Optimizing extraction parameters (temperature, time, pH, and salt content) and thermal desorption parameters (temperature and time) was performed by Face-Centered Central Design-response surface methodology (FCCD-RSM). The developed Zr-UiO-66-PDC@NTD method provides lower LODs, lower LOQs, acceptable repeatability and reproducibility, wider LDRs, and higher extraction efficiency. The results illustrated that the proposed method can be successfully applied as a simple, sensitive, solvent-free, fast, user-friendly, eco-friendly, and cost-effective drug-test for single-step headspace sampling and analysis of trace levels of the amphetamine compounds from the urine matrix of the employments, sports, poisoning diagnostics, and forensics.

## Supplementary Information

Supplementary Information.

## Data Availability

The datasets used and analyzed during the current study available from the corresponding author on reasonable request.
