# Supplementary material for: Urinary bio-monitoring of amphetamine derivatives by needle trap device packed with the zirconium-based metal–organic framework
Source: Sci Rep. 2022 Aug 11;12:13702. doi: 10.1038/s41598-022-17861-1 (PMC9372183; doi:10.1038/s41598-022-17861-1)
Supplement: Supplementary file 1 — Supplementary Information. [file 41598_2022_17861_MOESM1_ESM.docx]

**Urinary Bio-Monitoring of Amphetamine Derivatives by Needle Trap Device Packed with the Zirconium-Based Metal-Organic Framework**

Razzagh Rahimpoor^1^, Ali Firoozichahak ^*2^, Saber Alizadeh ^*3^, Davood Nematollahi^3^

*^1^Department of Occupational Health Engineering, Research Center for Health Sciences, School of Health, Larestan University of Medical Sciences, Larestan, Iran*

*^2^ Department of Occupational Health, Faculty of Health, Social Determinants of Health Research Center, Gonabad University of Medical Science, Gonabad, Iran*

*^3^Department of Chemistry, Bu-Ali-Sina University, Hamedan, Iran.*

**Corresponding authors:*

*1- Ali firoozichahak, Faculty Member, Department of Occupational Health, Faculty of Health, Gonabad University of Medical Sciences, Gonabad, Iran.*

*Email: a.firoozi@edu.umsha.ac.ir*

*2- Saber Alizadeh, Department of Chemistry, Bu-Ali-Sina University, Hamedan, Iran.*

*Email:* [*s.alizade66@yahoo.com*](mailto:s.alizade66@yahoo.com)


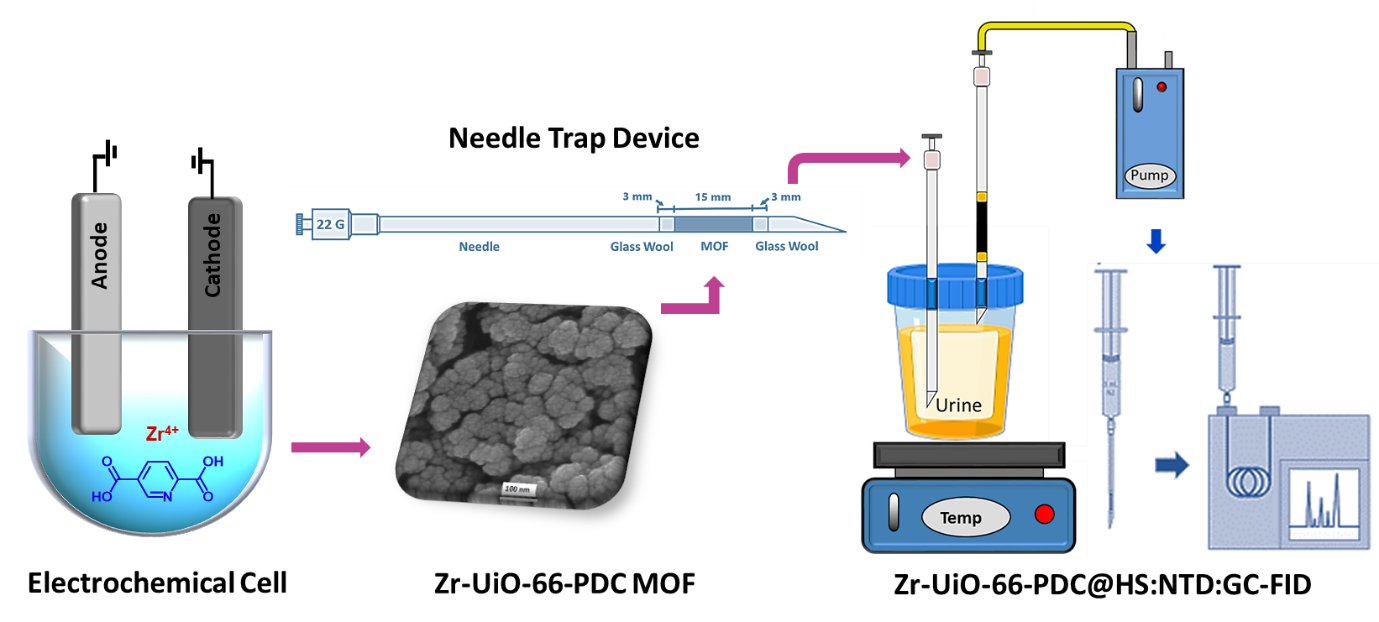


**Figure S1:** Schematic diagram of electrochemical synthesis of Zr-UiO-66-PDC MOF and headspace sampling and analysis of the amphetamine compounds by Zr-UiO-66-PDC@NTD technique

| **Table S1**  Analysis of variance (ANOVA) table for the optimization of desorption parameters of Amphetamine sampled with NTD: Zr-UIO-66-PDC | | | | | | |
| --- | --- | --- | --- | --- | --- | --- |
| **Source** | **Sum of Squares** | **Df^a^** | **Mean Square** | **F-Value** | **p-value**  **Prob > F** |  |
| Model | 3.624E+006 | 5 | 7.249E+005 | 11.45 | 0.0029 | significant |
| A-Temperature | 2.417E+006 | 1 | 2.417E+006 | 38.18 | 0.0005 |  |
| B-Time | 5.655E+005 | 1 | 5.655E+005 | 8.93 | 0.0203 |  |
| AB | 21462.25 | 1 | 21462.25 | 0.34 | 0.5786 |  |
| A2 | 4.479E+005 | 1 | 4.479E+005 | 7.08 | 0.0325 |  |
| B2 | 16684.78 | 1 | 16684.78 | 0.26 | 0.6235 |  |
| Residual | 4.431E+005 | 7 | 63294.81 |  | 0.0029 |  |
| Lack of Fit | 50444.44 | 3 | 16814.81 | 0.17 | 0.9105 | not significant |
| Pure Error | 3.926E+005 | 4 | 98154.80 |  |  |  |
| Cor Total | 4.067E+006 | 12 |  |  |  |  |
| R-Squared | 0.8811 |  |  |  |  |  |
| Adj R-Squared | 0.8533 |  |  |  |  |  |
| ^a^ df: Degree of freedom | | | | | | |

| **Table S2**  Analysis of variance (ANOVA) table for the optimization of desorption parameters of Fenfluramine sampled with NTD: Zr-UIO-66-PDC | | | | | | |
| --- | --- | --- | --- | --- | --- | --- |
| **Source** | **Sum of Squares** | **Df^a^** | **Mean Square** | **F-Value** | **p-value**  **Prob > F** |  |
| Model | 3.389E+005 | 5 | 67777.90 | 11.87 | 0.0026 | significant |
| A-Temperature | 2.388E+005 | 1 | 2.388E+005 | 41.83 | 0.0003 |  |
| B-Time | 49904.64 | 1 | 49904.64 | 8.74 | 0.0212 |  |
| AB | 1701.56 | 1 | 1701.56 | 0.30 | 0.6020 |  |
| A2 | 34487.65 | 1 | 34487.65 | 6.04 | 0.0436 |  |
| B2 | 1492.32 | 1 | 1492.32 | 0.28 | 0.6249 |  |
| Residual | 39959.50 | 7 | 5708.50 |  | 0.0026 |  |
| Lack of Fit | 4623.77 | 3 | 1541.26 | 0.18 | 0.9284 | not significant |
| Pure Error | 35335.73 | 4 | 8833.93 |  |  |  |
| Cor Total | 3.788E+005 | 12 |  |  |  |  |
| R-Squared | 0.8945 |  |  |  |  |  |
| Adj R-Squared | 0.8192 |  |  |  |  |  |
| ^a^ df: Degree of freedom | | | | | | |

| **Table S3**  Analysis of variance (ANOVA) table for the optimization of desorption parameters of Methamphetamine sampled with NTD: Zr-UIO-66-PDC | | | | | | |
| --- | --- | --- | --- | --- | --- | --- |
| **Source** | **Sum of Squares** | **Df^a^** | **Mean Square** | **F-Value** | **p-value**  **Prob > F** |  |
| Model | 6.638E+006 | 5 | 1.328E+006 | 49.06 | < 0.0001 | significant |
| A-Temperature | 3.893E+006 | 1 | 3.893E+006 | 143.85 | < 0.0001 |  |
| B-Time | 1.188E+006 | 1 | 1.188E+006 | 43.89 | 0.0003 |  |
| AB | 70740.04 | 1 | 70740.04 | 2.61 | 0.1500 |  |
| A2 | 1.117E+006 | 1 | 1.117E+006 | 41.27 | 0.0004 |  |
| B2 | 25377.70 | 1 | 25377.70 | 0.94 | 0.3651 |  |
| Residual | 1.894E+005 | 7 | 27063.12 |  | < 0.0001 |  |
| Lack of Fit | 62049.74 | 3 | 20683.25 | 0.65 | 0.6235 | not significant |
| Pure Error | 1.274E+005 | 4 | 31848.02 |  |  |  |
| Cor Total | 6.827E+006 | 12 |  |  |  |  |
| R-Squared | 0.9723 |  |  |  |  |  |
| Adj R-Squared | 0.9524 |  |  |  |  |  |
| ^a^ df: degree of freedom | | | | | | |

| **Table S4**  Analysis of variance (ANOVA) table for the optimization of extraction parameters of Amphetamine sampled with NTD: Zr-UIO-66-PDC | | | | | | |
| --- | --- | --- | --- | --- | --- | --- |
| **Source** | **Sum of**  **Squares** | **Df^a^** | **Mean**  **Square** | **F-Value** | **p-value**  **Prob > F** |  |
| Model | 3.690E+006 | 10 | 3.690E+005 | 24.68 | < 0.0001 | significant |
| A-Temperature | 1.648E+006 | 1 | 1.648E+006 | 110.26 | < 0.0001 |  |
| B-Time | 1.218E+006 | 1 | 1.218E+006 | 81.46 | < 0.0001 |  |
| C-Salt | 1.299E+005 | 1 | 1.299E+005 | 8.69 | 0.0083 |  |
| D-pH | 2.803E+005 | 1 | 2.803E+005 | 18.75 | 0.0004 |  |
| AB | 1.094E+005 | 1 | 1.094E+005 | 7.32 | 0.0140 |  |
| AC | 5076.56 | 1 | 5076.56 | 0.34 | 0.5669 |  |
| AD | 1.667E+005 | 1 | 1.667E+005 | 11.15 | 0.0034 |  |
| BC | 15190.56 | 1 | 15190.56 | 1.02 | 0.3261 |  |
| BD | 82512.56 | 1 | 82512.56 | 5.52 | 0.0298 |  |
| CD | 35062.56 | 1 | 35062.56 | 2.35 | 0.1421 |  |
| Residual | 2.840E+005 | 19 | 14949.46 |  |  |  |
| Lack of Fit | 2.181E+005 | 14 | 15580.49 | 1.18 | 0.4605 | not significant |
| Pure Error | 65912.83 | 5 | 13182.57 |  |  |  |
| Cor Total | 3.974E+006 | 29 |  |  |  |  |
| R-Squared | 0.9285 |  |  |  |  |  |
| Adj R-Squared | 0.8909 |  |  |  |  |  |
| ^a^ df: degree of freedom | | | | | | |

| **Table S5**  Analysis of variance (ANOVA) table for the optimization of extraction parameters of Fenfluramine sampled with NTD: Zr-UIO-66-PDC | | | | | | |
| --- | --- | --- | --- | --- | --- | --- |
| **Source** | **Sum of**  **Squares** | **Df^a^** | **Mean**  **Square** | **F-Value** | **p-value**  **Prob > F** |  |
| Model | 2.496E+006 | 10 | 2.496E+005 | 41.35 | < 0.0001 | significant |
| A-Temperature | 9.603E+005 | 1 | 9.603E+005 | 159.08 | < 0.0001 |  |
| B-Time | 8.649E+005 | 1 | 8.649E+005 | 143.27 | < 0.0001 |  |
| C-Salt | 1.464E+005 | 1 | 1.464E+005 | 24.25 | < 0.0001 |  |
| D-pH | 2.215E+005 | 1 | 2.215E+005 | 36.69 | < 0.0001 |  |
| AB | 70013.16 | 1 | 70013.16 | 11.60 | 0.0030 |  |
| AC | 11449.00 | 1 | 11449.00 | 1.90 | 0.1845 |  |
| AD | 1.067E+005 | 1 | 1.067E+005 | 17.67 | 0.0005 |  |
| BC | 22081.96 | 1 | 22081.96 | 3.66 | 0.0710 |  |
| BD | 52808.04 | 1 | 52808.04 | 8.75 | 0.0081 |  |
| CD | 39920.04 | 1 | 39920.04 | 6.61 | 0.0187 |  |
| Residual | 1.147E+005 | 19 | 6036.66 |  |  |  |
| Lack of Fit | 72512.31 | 14 | 5179.45 | 0.61 | 0.7834 | not significant |
| Pure Error | 42184.21 | 5 | 8436.84 |  |  |  |
| Cor Total | 2.611E+006 | 29 |  |  |  |  |
| R-Squared | 0.9561 |  |  |  |  |  |
| Adj R-Squared | 0.9329 |  |  |  |  |  |
| ^a^ df: degree of freedom | | | | | | |

| **Table S6**  Analysis of variance (ANOVA) table for the optimization of extraction parameters of Methamphetamine sampled with NTD: Zr-UIO-66-PDC | | | | | | |
| --- | --- | --- | --- | --- | --- | --- |
| **Source** | **Sum of**  **Squares** | **Df^a^** | **Mean**  **Square** | **F-Value** | **p-value**  **Prob > F** |  |
| Model | 6.433E+006 | 10 | 6.433E+005 | 42.81 | < 0.0001 | significant |
| A-Temperature | 2.494E+006 | 1 | 2.494E+006 | 165.96 | < 0.0001 |  |
| B-Time | 2.158E+006 | 1 | 2.158E+006 | 143.62 | < 0.0001 |  |
| C-Salt | 3.459E+005 | 1 | 3.459E+005 | 23.02 | 0.0001 |  |
| D-pH | 6.054E+005 | 1 | 6.054E+005 | 40.29 | < 0.0001 |  |
| AB | 1.515E+005 | 1 | 1.515E+005 | 10.08 | 0.0050 |  |
| AC | 50097.63 | 1 | 50097.63 | 3.33 | 0.0836 |  |
| AD | 2.628E+005 | 1 | 2.628E+005 | 17.49 | 0.0005 |  |
| BC | 54067.88 | 1 | 54067.88 | 3.60 | 0.0731 |  |
| BD | 1.716E+005 | 1 | 1.716E+005 | 11.42 | 0.0031 |  |
| CD | 1.403E+005 | 1 | 1.403E+005 | 9.34 | 0.0065 |  |
| Residual | 2.855E+005 | 19 | 15025.67 |  |  |  |
| Lack of Fit | 1.741E+005 | 14 | 12435.35 | 0.56 | 0.8208 | not significant |
| Pure Error | 1.114E+005 | 5 | 22278.54 |  |  |  |
| Cor Total | 6.719E+006 | 29 |  |  |  |  |
| R-Squared | 0.9575 |  |  |  |  |  |
| Adj R-Squared | 0.9351 |  |  |  |  |  |
| ^a^ df: degree of freedom | | | | | | |


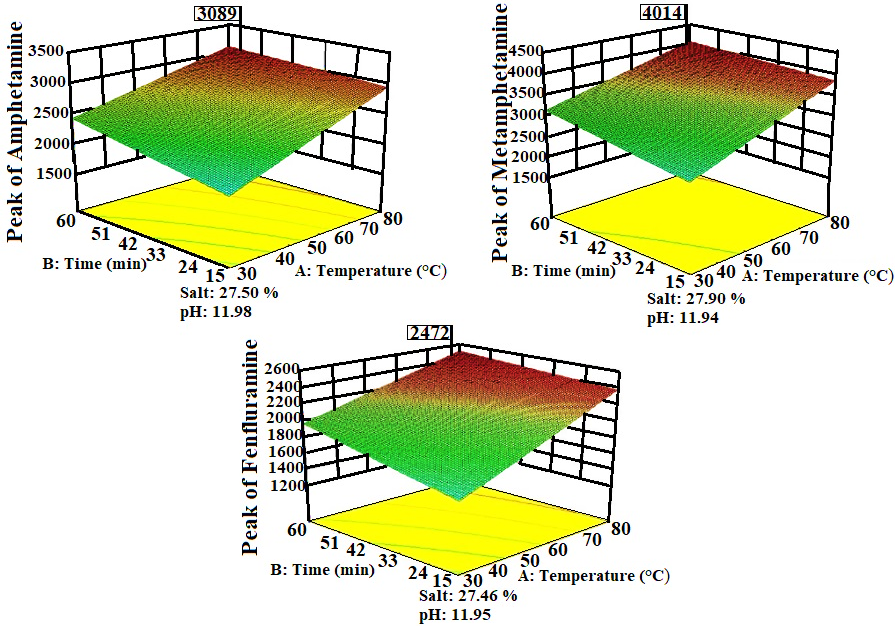


**Figure S2**. The effect of extraction parameters on the analysis performance of urinary amphetamine compounds by Zr-UiO-66-PDC@NTD method


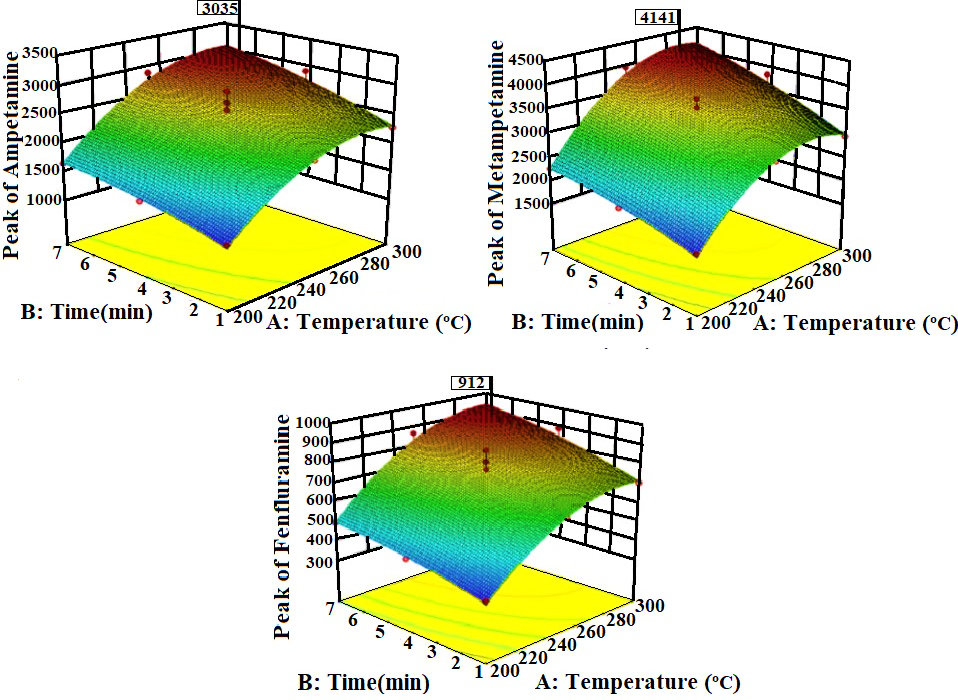


**Figure S3**. The effect of desorption parameters on the analysis performance of urinary amphetamine compounds by the Zr-UiO-66-PDC@NTD method


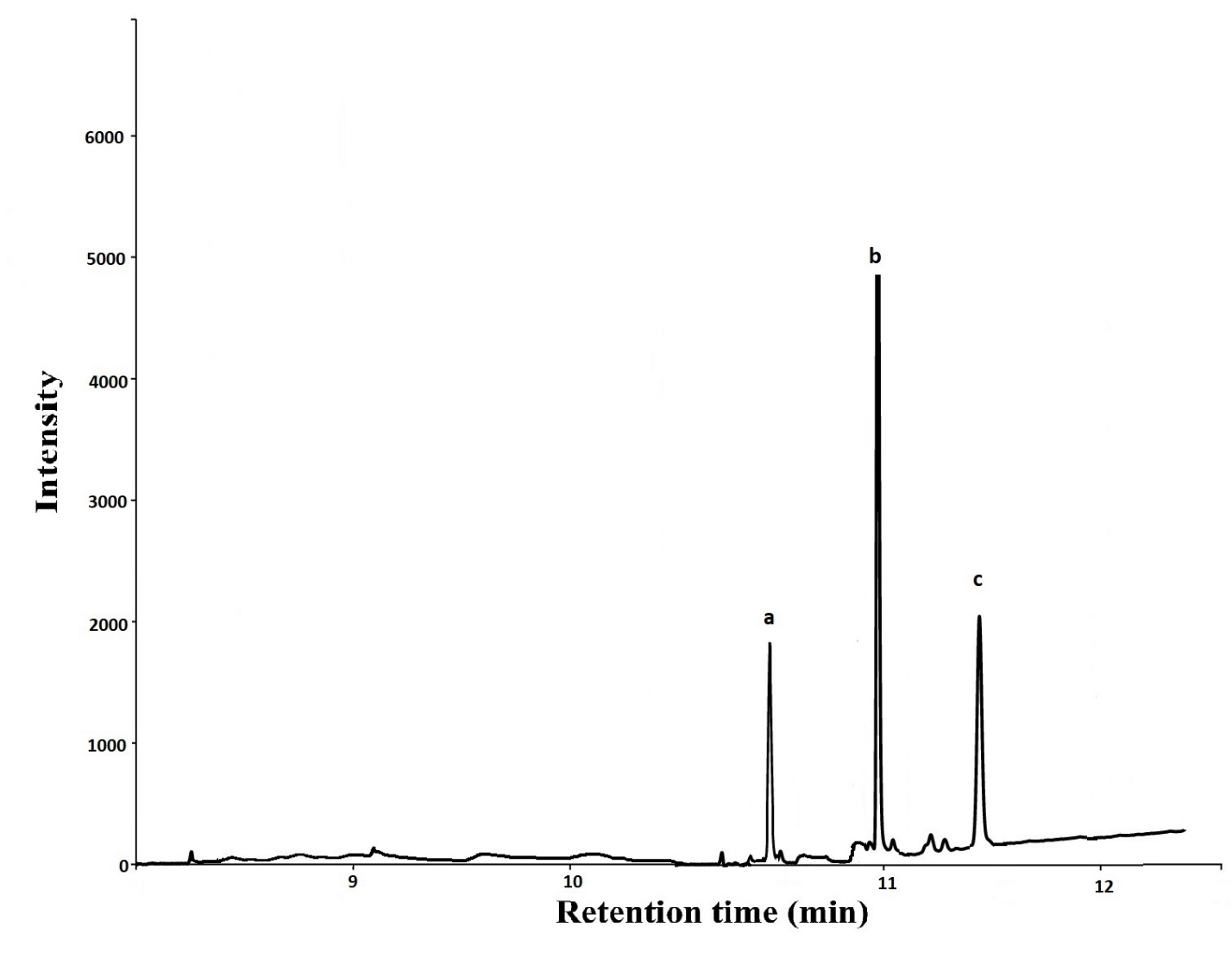


**Figure S4**. GC- FID chromatogram of amphetamine compounds in the real urine samples by proposed Zr- UiO-66-PDC: NTD method **a**: Amphetamine **b**: Methamphetamine **c**: Fenfluramine


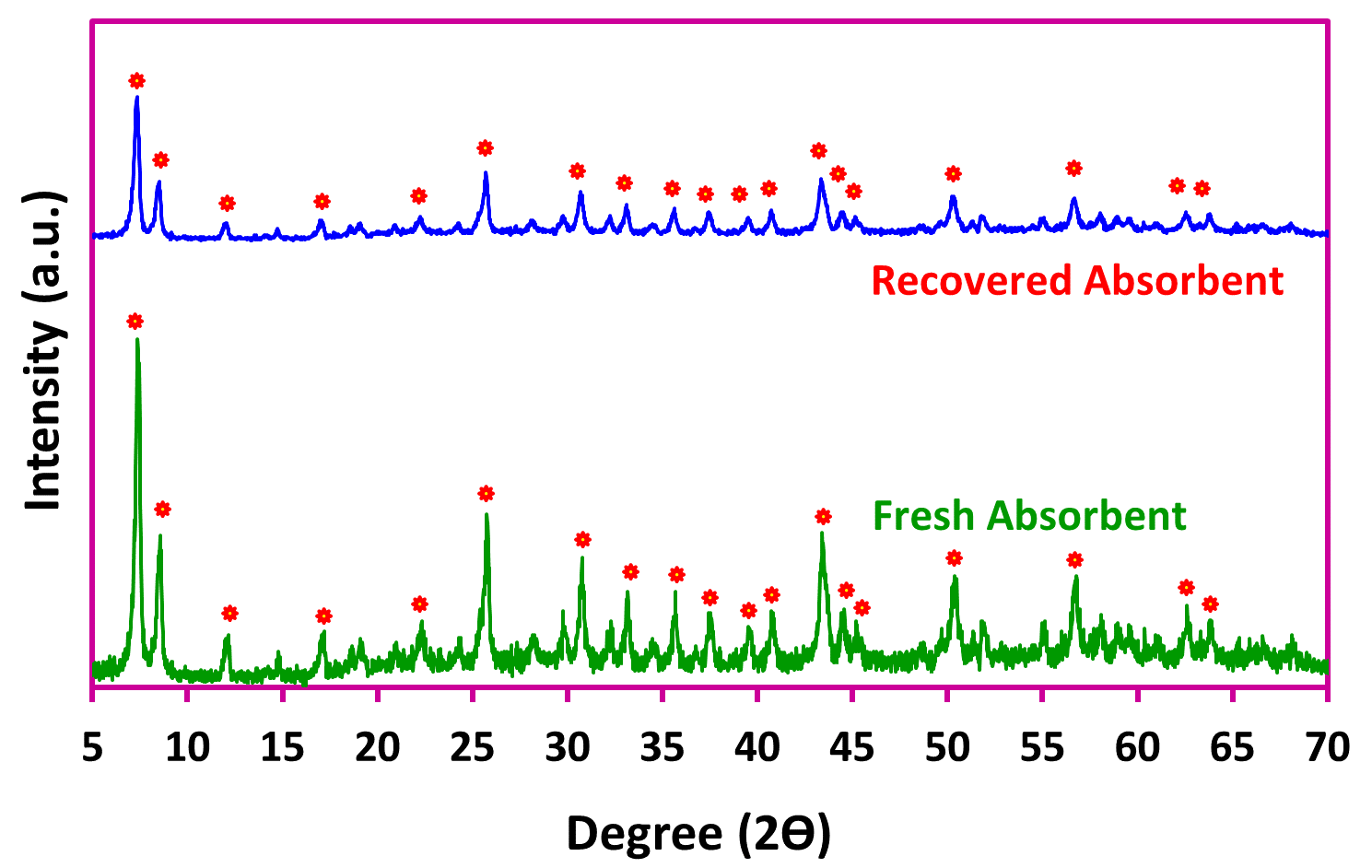


**Figure S5**. The PXRD pattern of the fresh and recovered Zr- UiO-66-PDC adsorbent in NTD system.


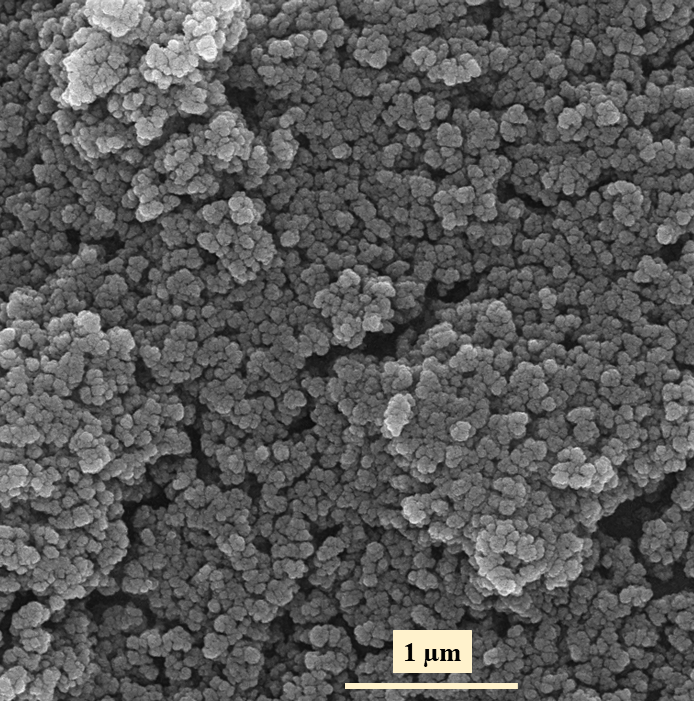


**Figure S6**. The FE-SEM image of the recovered Zr- UiO-66-PDC adsorbent in NTD system
